# Effects of Pectus Excavatum on the Spine of Pectus Excavatum Patients with Scoliosis

**DOI:** 10.1155/2017/5048625

**Published:** 2017-07-03

**Authors:** WeiHong Zhong, JinDuo Ye, JingJing Feng, LiYang Geng, GuangPu Lu, JiFu Liu, ChunQiu Zhang

**Affiliations:** ^1^Tianjin Key Laboratory of the Design and Intelligent Control of the Advanced Mechatronical System, Tianjin University of Technology, Binshuixi Road, No. 391, Tianjin 300384, China; ^2^Tianjin Key Laboratory for Control Theory & Applications in Complicated Systems, Tianjin University of Technology, Binshuixi Road, No. 391, Tianjin 300384, China; ^3^Military General Hospital of Beijing PLA, Beijing 100026, China

## Abstract

**Background:**

There is high risk in the correction surgery of pectus excavatum with scoliosis because of the lack of the correction mechanism of pectus excavatum with scoliosis. This study performed a comprehensive analysis about the impact that pectus excavatum had on scoliosis and elaborated its biomechanical mechanism in pectus excavatum patients with scoliosis.

**Methods:**

37 pectus excavatum patients were selected. According to age, Haller index of pectus excavatum, offset coefficient, vertical position, sternal torsion angle, and asymmetric index, 37 patients were, respectively, divided into 2 compared groups. The result was statistically calculated.

**Results:**

The scoliosis incidence and severity did not correlate with Haller index, offset coefficient, vertical position, sternal torsion angle, and asymmetric index of pectus excavatum, and there was no statistical significance between the two compared groups.

**Conclusions:**

The incidence and severity of scoliosis in PE patients with scoliosis have nothing to do with the geometric parameters of pectus excavatum but correlate with age. The scoliosis will aggravate with the increase of age. The heart may provide an asymmetric horizontal force to push the spines to the right. The mechanism of how the biomechanical factors exert influences on spines needs to be further investigated to keep the spine stable.

## 1. Background

Pectus excavatum (PE) is the most frequently observed congenital deformity of the chest which is characterized, in most cases, by a deep depression of the sternum in the anterior thoracic wall. There is a high percentage of scoliosis associated with PE [1, 2]. William Rainey Johnson reported that about more than 20% of PE patients had scoliosis [3]. In addition, surgical correction for PE has become more prevalent with the development of the minimally invasive Nuss procedure [4]. Due to the integrality of the anterior and posterior thorax, the Nuss procedure not only corrects concavity of the anterior region of the thorax through the placement of bars but also has dynamic effects on the spine in asymmetric PE [5], which means PE correction may be accompanied by the risk of scoliosis generation or aggravation. We need to investigate the relationship between PE and scoliosis and to find how PE affects the spine in PE patients, especially in PE patients with scoliosis.

Up to now, no comprehensive analysis about the impact that PE has on scoliosis has been performed, although precious research has involved correlation among scoliosis, age, and severity of PE [6]. Being the accompanying symptom or the postoperative complications, the effecting factors of PE on scoliosis and the relationship between scoliosis and age and so on have not been studied in the past. This study aims to analyze the influences that all the geometric parameters of the congenital PE have on scoliosis and to propose rational suggestions for studying out operation plans so as to offer essential help for surgeons to perform specific surgical procedures and it also tries to elaborate the biomechanical mechanisms of pectus excavatum.

## 2. Methods

### 2.1. Patients

The selection of patients was performed by referring to radiographic or computed tomographic images collected preoperatively. From patients of PE who underwent the Nuss procedure at the Military General Hospital of Beijing PLA from February 2009 to March 2014, a total of 37 patients (22 males and 15 females) with an asymmetry of the thorax and a mild to moderate deformity of the spine (with a Cobb angle ≤30°) were selected, and we excluded patients having platy thorax and barrel chest. The patients were between the age of 4 and 44 in this study.

### 2.2. Measurement

In this study, spiral computerized tomography scan was employed and preoperative CT scans of 37 patients were collected and saved in Digital Imagine and Communication in Medicine (DICOM) format. Then, the preoperative CT images were inputted into the medical image 3D reconstruction software Mimics10.01 (Materialise, Belgium), in which all radiological measurements were conducted.

Cobb angle was defined as a coronal plane deformity on anteroposterior plain radiographs to describe scoliosis. As a general rule, a Cobb angle of 10° is the minimum angulation for scoliosis.

**Figure 1:**
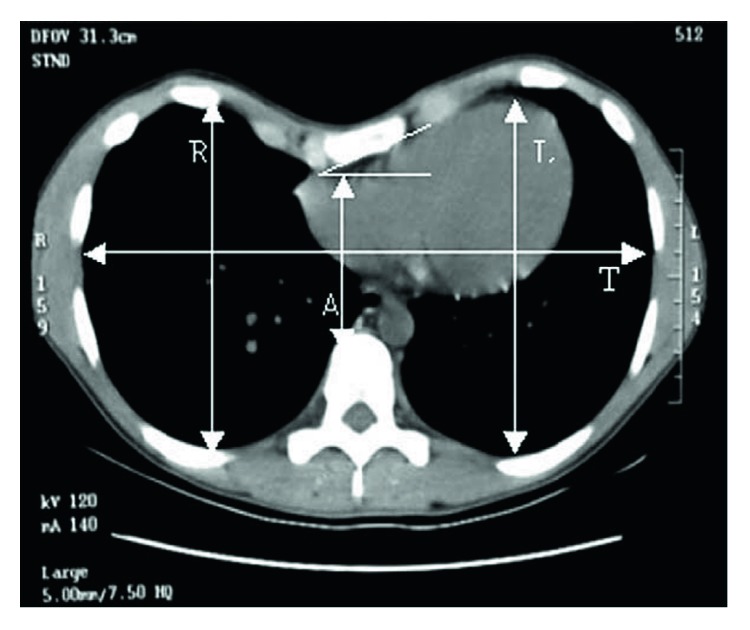
Demonstration of measurements made using Mimics on computer. The Haller index is calculated by T/A and asymmetry index by R/L×100. The sternal torsion angle is marked and represents moderate degree of torsion (+24.9°). All measurements were measured at maximum distances except for A, which was measured as the minimum distance between the anterior surface of the vertebral column and the deepest portion of the sternum.

Haller index (HI) was created in 1987 by Drs. Haller, Kramer, and Lietman [6]. The index is the ratio of the transverse diameter and the anteroposterior diameter is

(1) $$\begin{array}{c} \text{Haller index}=\frac{T}{A}\times 100, \end{array}$$

where *T* is the transverse diameter of the inside ribcage and *A* is the distance between the sternum and vertebrae (Figure 1). A normal Haller index is about 2.5. If the Haller index is greater than 3.25, the surgery is warranted [7]. The mean Haller index reported by Kelly et al. was 5.15 ± 2.32 [8].

For the analysis and investigation of the degree and different types of asymmetric deformity of the chest, symmetry index (SI) and sternal rotation angle have been widely used [9, 10]. Symmetric index is defined as the following ratio:

(2) $$\begin{array}{c} \text{Symmetric index}=\frac{R}{L}\times 100. \end{array}$$

The sternal torsion angle (STA) against the horizontal line was measured. The right-side depression of the chest wall, which indicates the counter clockwise twist of the sternum, is expressed as positive (Figure 1). Either the right- or left-side depression of PE, the sternal rotation angle that is less than 5°, greater than 5° but less than 15°, greater than 15° but less than 25°, and over 25° is regarded as symmetrical, mild, moderate and severe torsion, respectively [10].

**Figure 2:**
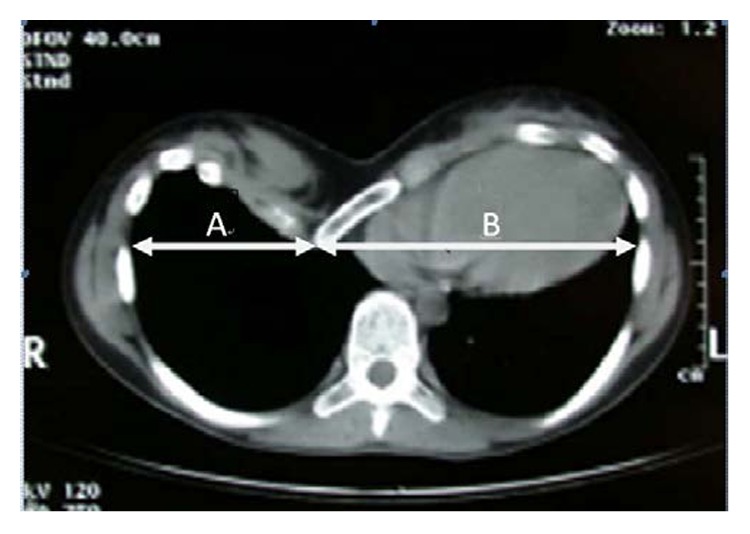
Demonstration of measurements using Mimics on computer. Offset coefficient calculated by A/B×100 is shown in the figure. This CT of a 12-year-old patient exhibited 57 of the offset coefficient; PE apex is on the right chest.

In addition, to investigate the degree of asymmetric deformity of PE in the horizontal direction, offset coefficient (OC) was defined to describe the excursion degree of the center of PE apex (Figure 2). The offset coefficient is a hundred times the ratio of the distance A and distance B; its mathematical relationship is shown as follows:

(3) $$\begin{array}{c} \text{Offset coefficient}=\frac{A}{B}\times 100, \end{array}$$

where *A* is the distance between the left chest wall and the apex and *B* is the distance between the apex and the right chest wall. A normal offset coefficient is 1. If it is not equal to 1, meaning that PE is asymmetric and its apex offsets to the left or the right side. If it is more than 1, the PE apex is on the left chest; if less than 1, the PE apex is on the right chest. The greater the absolute value of the offset coefficient away from 1 is, the more severe the excursion of PE apex is.

### 2.3. Groups

37 patients were divided into two age groups preoperatively—the child group (*n* = 15) and the adult group (*n* = 22). The ages were no more than 18 years old in the child group and older than 18 years old in the adult group.

According to the severity of PE, 37 patients were divided into two Haller index groups preoperatively—the mild group (*n* = 5) and the severe group (*n* = 32). The Haller indexes in the mild group were greater than 3.25 and less than 3.5, while the Haller indexes in the severe group were greater than or equal to 3.5.

37 patients were divided into 2 offset coefficient groups: the mild group (*n* = 7), in which offset coefficient was less than or equal to 10; the sever group (*n* = 30), in which offset coefficient was more than 10.

37 patients were divided into 2 sternal torsion angle groups: the mild group (*n* = 17), in which the sternal torsion angle was less than 25°; the sever group (*n* = 20), in which the sternal torsion angle was equal to or more than 25°.

### 2.4. Statistical Methods

For statistical calculations, SPSS Version 20.0 for Windows (SPSS Inc., IBM Company, Chicago, IL) was used.

### 2.5. Ethical Committee

This study has obtained approval by the school of mechanical engineering, Tianjin University of Technology and Military General Hospital of Beijing PLA. There is no conflict of interest to be declared.

## 3. Results and Discussion

### 3.1. Scoliosis and Age

The results of the scoliosis incidence in the age groups were shown in Table 1. 28 (75.68%) of the 37 patients had scoliosis with a Cobb angle greater than 10°.We found that 20 (71.42%) out of the 28 patients with a Cobb angle greater than 10° were from adults. The cases of scoliosis distributed in the adult group were with a percentage of 90.91%, and the incidence of scoliosis was 53.33% in children less than 18 years. The computed result (*p* < 0.05) shows that there is a statistically significant difference between the two age groups, suggesting that age is correlated with scoliosis incidence.

**Figure 3:**
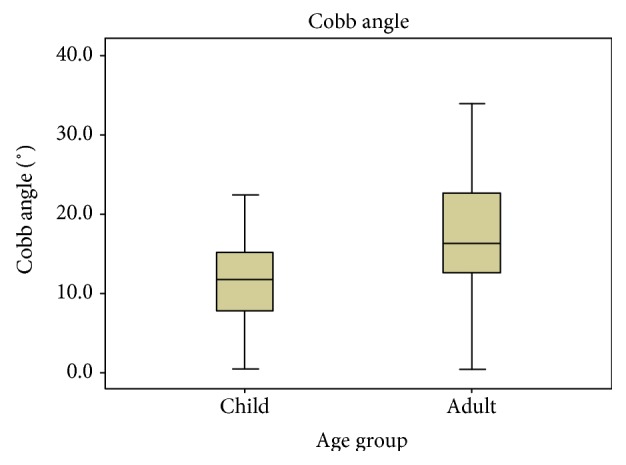
Cobb angles of the two age groups. Significant difference was found between the child and adult groups (*p* < 0.05).

Further statistics (*p* = 0.044) indicated that there was also a statistically significant difference between the two age groups in the severity of scoliosis. The Cobb angles in the adult group were obviously higher than those in the child group (Figure 3).

In this study, the incidence of scoliosis in the adult group was 90.91% (Table 1), which is much higher than that in the child group (53.33%); the severity of scoliosis correlated with age. It is well known that the deformity of PE, including the depth and area of depression and degree of sternal twist causing asymmetry of the chest, progresses as the patients grow [10]. According to the biomechanical principle that deformity results from internal force, generation and change of horizontal internal force will result in the occurrence and change (including improvement or aggravation) of scoliosis. We speculate that the scoliosis deformity caused by internal force which the PE exerted on the thoracic cage progresses with age. The reason may be that as the patients grow older, their bones become more calcified, their costal cartilages become more brittle and more ossified, the internal horizontal force generated by PE pushes the heart more greatly, and the counterforce generated by the heart pushes the spine [6]; hence, the scoliosis becomes more severe.

### 3.2. Scoliosis and Haller Index

With regard to the severity of the PE, 25 (89.29%) of the 28 scoliosis patients (Cobb  angle > 10°) were from the severe HI group (HI > 3.5), in which the incidence of scoliosis was 78.13%, while 3 scoliosis cases were from the mild HI group (3.25 < HI ≤ 3.5), in which the incidence of scoliosis was 60.00%. The computed result (*p* = 0.105) shows that there is no statistic significant difference between the mild HI group and the severe HI group (Table 2).

Further statistics (*p* = 0.117) indicated that there was also no statistically significant difference between the two Haller index groups in the severity of scoliosis.

In this study, the incidence and the severity of scoliosis have nothing to do with the Haller index of PE. In the view of mechanics principle, the severity of PE described in the form of Haller index has no impact on the internal force exerted on the thoracic cage, except on the depth of the concavity. It cannot break the balance between PE and the spine. So, it cannot change the incidence and the severity of scoliosis.

### 3.3. Scoliosis and Offset Coefficient

The results of the scoliosis incidence in offset coefficient groups were shown in Table 3. 23 (82.14%) of the 28 scoliosis cases were from the severe OC group (OC > 10), in which the incidence of scoliosis was 76.67%, while 5 scoliosis cases were from the mild OC group (OC ≤ 10), in which the incidence of scoliosis was 66.67%. There is no statistic significant difference between the mild OC group and the severe OC group (*p* > 0.05, Table 3).

Further statistics (*p* = 0.813) indicated that there was also no statistically significant difference between the two offset coefficient groups in the severity of scoliosis.

Offset coefficient describes the excursion degree of PE apex in the horizontal direction, which represents the horizontal parameter of the PE position. Different offset coefficients contribute nothing to the generation and change of the horizontal internal force which is exerted to the spine. So, it can not have any effect on the incidence and severity of scoliosis.

### 3.4. Scoliosis and Vertical Position of PE

The cases of vertical position distribution of PE in the high group (*n* = 19) and the low group (*n* = 18) were close to equal (Table 4), and the scoliosis incidence in every group was 88.89% and 63.16%, respectively. The computed result indicated that the incidence and severity of scoliosis did not correlate with the vertical position of PE (*p* > 0.05), and there was no statistical significance between the high group and the low group in the incidence and severity of scoliosis (*p* > 0.05).

The results of the scoliosis direction in different vertical position of PE group were shown in Table 5. In 20 (54.05%) of 37 patients of PE, their scoliosis bent to the right, 8 (21.62%) patients bent to the left, and 9 (24.32%) patients had no scoliosis or their Cobb angles were no more than 10°. The computed result indicated that the direction of scoliosis correlated with the vertical position of PE (*p* = 0.024), and there was a statistical significance in the direction of scoliosis between the high and low groups (*p* < 0.05).

**Figure 4:**
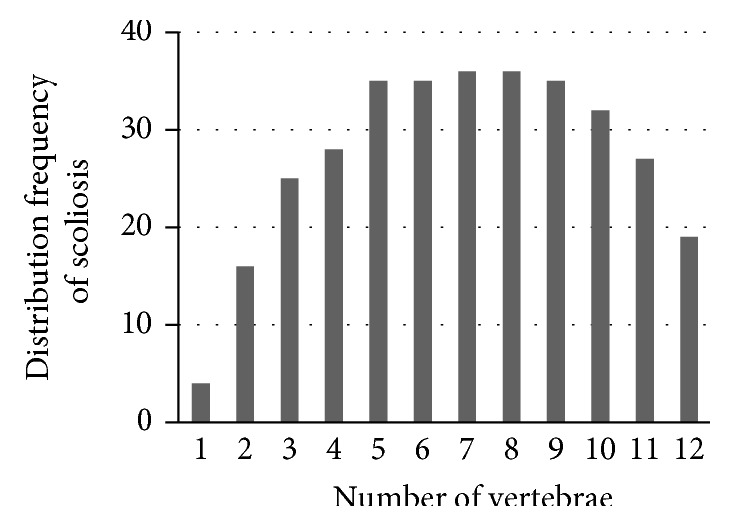
Distribution of the scoliosis vertical position in 37 patients in the form of thoracic vertebra number.

**Figure 5:**
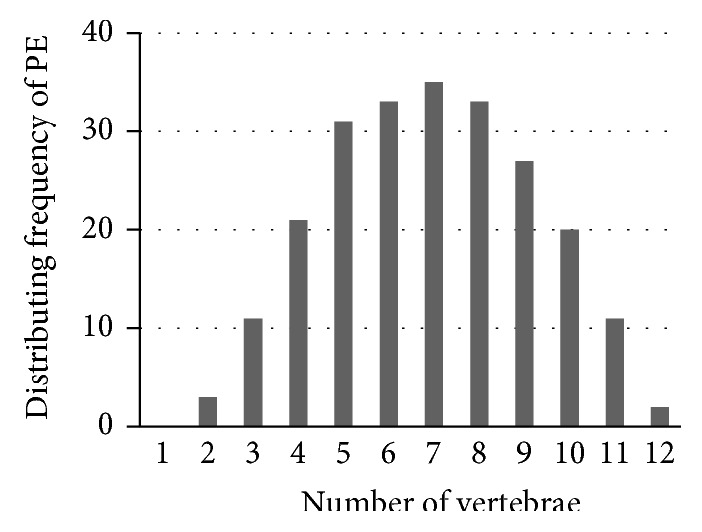
Distribution of the PE vertical position in 37 patients in the form of thoracic vertebra number.

Vertical position distribution describes the parameter in the vertical direction of PE, and it does not change the horizontal internal force which can make the thoracic vertebra stable in the horizontal direction. So, it has nothing to do with the incidence and the severity of scoliosis. But different vertical positions can affect the direction of scoliosis. We noticed that scoliosis is almost located at the same horizontal level with PE (Figures 4 and 5). Scoliosis in 37 patients mainly distributed from the 3rd to 11th thoracic vertebrae. 20 (71.43%) of the 28 PE with scoliosis patients bent to the right, and their PE position is mainly located in the scope of the 4th to 10th thoracic vertebrae where the heart was located. PE pushes the heart to the left, with the transformation of the ribs and costa cartilages; the internal counter force generated by the heart will push the thoracic vertebrae to the right, which means the heart may provide an asymmetric horizontal force to push the spines to the right in pectus excavatum patients with scoliosis. Thus, most of the scoliosis patients bent to the right, which verified that the heart contributes to the internal force causing the spine bent to the right [6].

### 3.5. Scoliosis and Sternal Torsion Angle and Symmetric Index

The results of the scoliosis incidence in two sternal torsion angle groups were shown in Table 6. We found that 20 (54.05%) of the 37 scoliosis cases were from the severe STA group (STA ≥ 25°), in which the incidence of scoliosis was 85.00%, while 17 (45.94%) scoliosis cases were from the mild STA group (STA < 25°), in which the incidence of scoliosis was 64.71%. There is no statistically significant difference between the mild STA group and the severe STA group (*p* > 0.05, Table 6).

Further statistics (*p* > 0.05) indicated that there was also no statistically significant difference between the two STA groups in the severity of scoliosis.

The results of the scoliosis incidence in the two symmetric index groups were shown in Table 7. Only 9 (32.14%) of the 28 scoliosis cases were in the severe SI group (SI ≥ 1.05 or SI ≤ 0.95), in which the incidence of scoliosis was 60.00%, while 19 (67.86%) of the scoliosis cases were from the mild SI group (STA < 25°), in which the incidence of scoliosis was 686.36%. There is no statistically significant difference between the SI mild group and the SI severe group (*p* > 0.05, Table 7).

Further statistics (*p* > 0.05) indicated that there was also no statistically significant difference between the two symmetric index groups in the severity of scoliosis.

Sternal torsion angle and symmetric index describe the asymmetric degree of the chest, and they do not correlate with the incidence and severity of scoliosis because they cannot break the balance of the horizontal internal force between PE and scoliosis. Hence, they devote nothing to the generation and change of the horizontal internal force and have no effect on the incidence and severity of scoliosis.

## 4. Conclusions

The incidence and severity of scoliosis in PE patients with scoliosis correlate with age. The scoliosis will aggravate with the increase of age, which suggests that once the scoliosis is diagnosed, treatment should be conducted as soon as possible.

The incidence and severity of scoliosis do not correlate with the Haller index, offset coefficient, vertical position of pectus excavatum, asymmetric index, and sternal torsion angle, which means that the severity of pectus excavatum and the horizontal and vertical positions of pectus excavatum have nothing to do with the geometric parameters of PE and do not have any effects on the incidence and severity of scoliosis. Namely, three-dimensional positional parameters and the symmetry of pectus excavatum in the human chest have no impact on scoliosis.

The heart may provide an asymmetric horizontal force to push the spines to the right, which means the mechanical factor may be the pathogenesis in pectus excavatum patients with scoliosis.

## Figures and Tables

**Table 1:** Age distribution of patients with a Cobb angle greater than 10°.

	Child group ≤18 Y (*n* = 15)	Adult group ≥18 Y (*n* = 22)	*p*
Scoliosis case (100%)	8 (53.33%)	20 (90.91%)	0.017 < 0.05

**Table 2:** Haller index distribution of patients with a Cobb angle greater than 10°.

	Mild group 3.2 < HI < 3.5 (*n* = 5)	Severe group HI ≥ 3.5 (*n* = 32)	*p*
Scoliosis case (100%)	3 (60%)	25 (78.13%)	0.105 > 0.05

**Table 3:** Offset coefficient distribution of patients with a Cobb angle greater than 10°.

	Mild OC group OC ≤ 10 (*n* = 7)	Severe OC group OC > 10 (*n* = 30)	*p*
Scoliosis case (100%)	5 (66.67%)	23 (76.67%)	1.000 > 0.05

**Table 4:** Vertical position distribution of pectus excavatum of patients with a Cobb angle greater than 10°.

	High group 1–6 (*n* = 18)	Low group 7–12 (*n* = 19)	*p*
Scoliosis case (100%)	16 (88.89%)	12 (63.16%)	1.000 > 0.05

**Table 5:** Vertical position distribution of patients with different scoliosis directions.

	High group 1–6 (*n* = 18)	Low group 7–12 (*n* = 19)	*p*
Scoliosis bent to the right (100%)	13 (72.22%)	7 (36.84%)	0.027 < 0.05
Scoliosis bent to the left (100%)	3 (16.67%)	5 (26.32%)	
No scoliosis	2 (11.11%)	7 (36.84%)	

**Table 6:** Sternal torsion angle distribution of pectus excavatum of patients with a Cobb angle greater than 10°.

	Mild group STA < 25° (*n* = 17)	Severe group STA ≥ 25° (*n* = 20)	*p*
Scoliosis case (100%)	11 (64.71%)	17 (85.00%)	0.251 > 0.05

**Table 7:** Asymmetric index distribution of pectus excavatum of patients with a Cobb angle greater than 10°.

	Mild group 0.95 < SI < 1.05 (*n* = 22)	Severe group SI ≥ 1.05 or SI ≤ 0.95 (*n* = 15)	*p*
Scoliosis case (100%)	19 (86.36%)	9 (60.00%)	0.118 > 0.05

## Abbreviations

PE: Pectus excavatum
CT: Computed tomography
DICOM: Digital Imaging and Communications in Medicine
HI: Haller index
OC: Offset coefficient
STA: Sternal torsion angle
AI: Asymmetric index.
